# Block Copolymers Based on Ethylene Glycol, Glycidol and β-Butyrolactone with Tunable Thermal Properties, Solubility, and Hydrolytic Degradation

**DOI:** 10.3390/ma19122467

**Published:** 2026-06-09

**Authors:** Marcelina Bochenek, Natalia Oleszko-Torbus, Agnieszka Kowalczuk, Wojciech Wałach

**Affiliations:** Centre of Polymer and Carbon Materials, Polish Academy of Sciences, M. Curie-Skłodowskiej 34, 41-819 Zabrze, Poland; noleszko@cmpw-pan.pl (N.O.-T.); akowalczuk@cmpw-pan.pl (A.K.); wwalach@cmpw-pan.pl (W.W.)

**Keywords:** block copolymer, tunable materials, poly(β-butyrolactone)

## Abstract

We report di- and triblock copolymers that combine hydrophilic polyethers—poly(ethylene glycol) monomethyl ether (mPEG) and polyglycidol (PGl)—with a hydrophobic, degradable polyester, poly(β-butyrolactone) (P(β-BL)). A mild hydrolysis method was developed to selectively remove acetal protecting groups from poly(ethoxy ethyl glycidyl ether) (PEEGE) without cleaving the β-butyrolactone polyester backbone, enabling the preparation of PGl-b-P(β-BL) and mPEG-b-PGl-b-P(β-BL) block copolymers. Thermal analysis revealed that the glass transition temperatures (*T*_g_) of the copolymers could be tuned by varying block composition and length. Diblock copolymers containing the PGl segment were amorphous, with *T*_g_ values ranging from −2.7 to −19.9 °C. The presence of an mPEG segment in the triblock copolymers resulted in a further decrease in *T*_g_, reaching values between −32.3 and −38.9 °C. Solubility and water affinity studies demonstrated that incorporation of hydrophilic polyether blocks enhances copolymer–solvent interactions, leading to increased wettability of the polymer-coated surface. The water contact angle for films formed from PGl-b-P(β-BL) decreased to 53 °C, while for mPEG-b-PGl-b-P(β-BL) copolymers, it was further reduced to 43 °C compared with the hydrophobic P(β-BL) film. Hydrolytic degradation experiments showed accelerated cleavage of the P(β-BL) segment in copolymers containing hydrophilic blocks compared to the P(β-BL) homopolymer, which is attributed to increased water accessibility and surface hydrophilicity. The most pronounced decrease in molar mass, reaching at least 50% relative to the initial non-degraded sample, was observed for the diblock copolymers, whereas the P(β-BL) sample showed only a marginal weight reduction of a few percent. Overall, this study demonstrates that the combination of hydrophilic mPEG and PGl blocks with P(β-BL) enables the design of block copolymers with tunable thermal properties, solubility, and degradation behavior, offering potential for a wide range of applications.

## 1. Introduction

Currently, materials used in medical and pharmaceutical applications, such as biodegradable implants, surgical sutures, drug delivery systems and tissue engineering, are predominantly based on biodegradable polymers that must meet strict biocompatibility and functional requirements. A special class of polymers applied in these fields is represented by polyhydroxyalkanoates (PHAs), a family of biodegradable polymers based on aliphatic polyesters [1,2,3,4,5,6]. Among them, poly(3-hydroxybutyrate) (PHB) is one of the important and widely studied biodegradable polyesters belonging to the PHA group [1,4]. PHB is naturally produced by bacteria as isotactic poly([R]-3-hydroxybutyrate) and serves as a carbon and energy storage material in microbial cells [7,8,9,10]. Its natural origin, biocompatibility and biodegradability make PHB a particularly promising material for medical applications. Moreover, the ultimate degradation product of PHB—hydroxybutyrates—are naturally present in living organisms, including mammalian blood [11], further supporting its suitability for biomedical use.

Despite these advantages, bacterially produced PHB is highly crystalline, brittle and hydrophobic; moreover, its degradation occurs in an uncontrolled manner [12,13]. Consequently, numerous studies have focused on developing strategies to reduce or eliminate its crystallinity, decrease its hydrophobicity, and introduce reactive functional groups into the polymer chain [1,3]. The most common approach to modifying bacterial PHB involves the incorporation of hydrophilic segments into the PHB backbone using various strategies, such as transesterification [14,15,16], “click” reactions [17,18,19], isocyanate chemistry [20,21,22,23], or atom transfer radical polymerization [24,25,26]. Using these approaches, various hydrophilic segments have been introduced, such as poly(N-isopropylacrylamide) [24], poly(2-(dimethylamino)ethyl methacrylate) [25,26] and poly(ethylene glycol) (PEG) [27,28,29,30]. Among hydrophilic polymers, PEG is the most commonly used for the modification of PHB-based materials due to its excellent biocompatibility and hydrophilicity, as PEGylation is highly desired for the improvement of the pharmacokinetic properties of therapeutic systems [31,32,33]. To overcome the intrinsic crystalline nature of bacterial PHB, polymerization of its synthetic analogue, β-butyrolactone (β-BL), has been applied [28,30,34,35]. Synthetic P(β-BL) exhibits the same structure as bacterial PHB and offers the advantages of an amorphous character and control over molar mass *via* controlled anionic polymerization [36]. Nevertheless, similar to bacterial PHB, P(β-BL) is hydrophobic and therefore typically functionalized through the incorporation of hydrophilic comonomers, with PEG being the most commonly used for this purpose. The key parameters investigated in modified PHB and P(β-BL) include glass transition temperature (*T*_g_), degradation rate, and solubility, properties that are crucial, especially to bio-related applications. A notable example of this type of modification is presented in the work of Li and co-workers [22], who synthesized PHB-based block copolymers by combining a hydrophobic PHB segment with a hydrophilic PEG segment using hexamethylene diisocyanate (HDI) as a coupling agent. Thermogravimetric analysis demonstrated the improved thermal stability of the resulting copolymers compared to the corresponding homopolymers. Furthermore, it was shown that the incorporation of PEG into the PHB chain significantly enhanced the wettability of the polymer-coated surface. In another study [37], block copolymers synthesized using HDI, composed of hydrophobic PHB, hydrophilic PEG, and thermosensitive poly(propylene glycol) PPG, formed hydrogels that degraded in phosphate buffer *via* random cleavage of PHB ester bonds into 3-hydroxybutyric acid and short oligomers. The protein release profiles of the copolymer hydrogels showed that the release rate was controlled by varying the composition of the PHB-PEG-PPG or by adjusting the concentration of the copolymer in the hydrogels. Jun Li et al. [30] prepared a PEG-based macroinitiator through TEMPO-mediated oxidation to initiate β-BL polymerization, yielding triblock copolymers consisting of a central PEG block and P(β-BL) side segments. Thermal analysis revealed the good compatibility of the PEG and P(β-BL) segments. Another example [35] described the synthesis of amphiphilic P(β-BL)-PEG-P(β-BL) triblock copolymers *via* tin(II) octanoate-catalyzed ring-opening polymerization, which were further formulated into core–shell nanoparticles and evaluated as drug delivery carriers, exhibiting accelerated degradation and controlled release of a hydrophobic compound during degradation of the P(β-BL) block. All the abovementioned examples demonstrate the synthesis of block copolymers combining hydrophobic PHB or P(β-BL) segments with hydrophilic PEG blocks, and studies show how structural changes mainly affect their thermal properties and degradation behavior. To date, however, copolymers incorporating PGl as a hydrophilic block have not been investigated. This is particularly important in view of the unique properties of PGl. In contrast to PEG, PGl contains pendant hydroxyl groups that allow for further chemical modification, significantly enhancing the functional potential of the resulting copolymers. Moreover, the presence of hydroxyl functionalities may promote hydrogen-bond-mediated interactions with surrounding aqueous environments, which can influence hydration behavior and interfacial properties. Hydroxyl groups also provide convenient sites for the attachment of bioactive ligands, drugs, or targeting moieties, making PGl-containing copolymers especially attractive for advanced biological and medical applications. Additionally, PGl has attracted increasing attention as an alternative hydrophilic segment due to concerns associated with PEG, including growing reports of immunogenic responses related to this polymer.

In this work, we address this gap by investigating the properties of diblock PGl-b-P(β-BL) and triblock PEG-b-PGl-b-P(β-BL) copolymers prepared using a previously developed method [38]. Importantly, this strategy represents the first use of PGl as a hydrophilic block in these systems. We investigate how the connection of hydrophilic mPEG (*M*_n SEC-MALLS_ = 2200 g/mol) and PGl segments with hydrophobic P(β-BL) affects solubility, water affinity, and thermal properties, as well as their influence on the hydrolytic degradation of the polyester block. In addition, we report, for the first time, an efficient deprotection strategy for removing acetal groups from the PEEGE block without degradation of the P(β-BL) segment. This approach is especially valuable, as it provides a reliable method to access functional PGl-containing copolymers while preserving the integrity of the hydrophobic polyester block. Thermal analysis demonstrated that the glass transition temperatures of the synthesized copolymers could be effectively tailored by adjusting the composition and relative lengths of the individual blocks, indicating the strong dependence of the thermal properties on the copolymer architecture. Due to the known biocompatibility of the components (PGl, PEG, and P(β-BL) blocks), the developed copolymers are promising candidates for biomedical applications, particularly as biodegradable drug delivery systems and tissue engineering materials. Their tunable amphiphilic character, which significantly influences the degradation rate of the polyester blocks, allows the material properties to be tailored to specific applications and adapted to physiological conditions. By systematically studying the structure–property relationships in these systems, our work provides insights that may guide the design of multifunctional, degradable copolymers with tunable physicochemical properties. Compared with similar copolymers reported in the literature, the main advantage of the developed materials is their precisely controlled copolymer structure, which enables a wide range of adjustments to their properties, particularly the hydrophilic–hydrophobic balance, while retaining hydroxyl groups that can be readily used for the modification and immobilization of biologically active substances. Since the copolymers described in this work have not previously been reported in the literature, a comprehensive characterization was performed. The structure of the copolymers was determined using ^1^H NMR spectroscopy; the molar masses were determined by size-exclusion chromatography with multi-angle laser light scattering (SEC-MALLS), a technique widely employed for the absolute determination of polymer molar masses. Thermal properties were investigated by differential scanning calorimetry (DSC), which is commonly used to determine glass transition temperatures and evaluate phase behavior in polymeric materials. Contact angle measurements, a standard method for assessing the hydrophilic–hydrophobic balance and surface wettability, were used to evaluate the affinity of the copolymer surfaces for water. Degradation studies were conducted according to the water solubility of the investigated copolymers. For water-insoluble materials, degradation was monitored gravimetrically, whereas for water-soluble or water-dispersible copolymers, degradation was followed by monitoring changes in molar mass using the SEC-MALLS technique. In addition, scanning electron microscopy (SEM) was employed to examine the surface morphology of copolymer films before and during degradation, enabling visualization of degradation-induced structural changes. Similar characterization approaches have been successfully applied to PEG-containing degradable polyesters and related copolymer systems [21,30,37,39,40,41].

## 2. Materials and Methods

### 2.1. Materials

β-butyrolactone (β-BL) (98%, Sigma Aldrich, St. Louis, MO, USA) was dried over calcium hydride (CaH_2_) (95%, Sigma Aldrich, St. Louis, MO, USA), distilled twice under reduced pressure (45 °C, 7 mbar) in argon atmosphere, then purified over potassium permanganate (KMnO_4_) (Avantor Performance Materials Poland S.A., Silesian Voivodeship, Gliwice, Poland), distilled using high vacuum line to the ampoule, stored under CaH_2_, and distilled prior to used. Glycidol with protected hydroxyl groups—1-ethoxy ethyl glycidyl ether (EEGE)—was synthesized from 2,3-epoxy-1-propanol (glycidol) (96%, Sigma Aldrich, St. Louis, MO, USA) and ethyl vinyl ether (99%, Sigma Aldrich, St. Louis, MO, USA) in reaction catalyzed by *p*-toluenesulfonic acid (98.5%, Sigma Aldrich, St. Louis, MO, USA) [42]. EEGE was stored under CaH_2_ and distilled prior to use. Poly(ethylene glycol) methyl ether (mPEG) (*M*_n SEC-MALLS_ = 2200 g/mol) (Tokyo Chemicals Industry Co., Tokyo, Japan) was dissolved in tetrahydrofuran (THF) (analytical grade, Avantor Performance Materials Poland S.A., Silesian Voivodeship, Gliwice, Poland), filtered and precipitated in n-hexane (for HPLC, Avantor Performance Materials Poland S.A., Silesian Voivodeship, Gliwice, Poland) and then dried under high vacuum. An mPEG-based macroinitiator was obtained according to the procedure described by Dworak et al. [43]. Succinic anhydride (99%, Sigma Aldrich, St. Louis, MO, USA) was sublimated and stored under an argon atmosphere. Tetrabutylammonium bromide (TBA Br) (Sigma Aldrich, St. Louis, MO, USA) and tetrabutylammonium acetate (97%, Sigma Aldrich, St. Louis, MO, USA) were dried under reduced pressure for 48 h and stored in an ampoule under an argon atmosphere. Cesium hydroxide monohydrate (CsOH·H_2_O) (Sigma Aldrich, St. Louis, MO, USA) and potassium *tert*-butoxide (*t*-BuOK) (99%, Sigma Aldrich, St. Louis, MO, USA) were used as received. THF for polymerization (analytical grade, Avantor Performance Materials Poland S.A., Silesian Voivodeship, Gliwice, Poland) was dried over CaH_2_, distilled, and refluxed over Na/K alloy immediately before use. Dimethyl sulfoxide (DMSO) (for HPLC, Avantor Performance Materials Poland S.A., Silesian Voivodeship, Gliwice, Poland) was dried over CaH_2_, distilled, dried over barium oxide (BaO) (97%, Sigma Aldrich, St. Louis, MO, USA), and fractionated twice under reduced pressure. Benzene (analytical grade, Sigma Aldrich, St. Louis, MO, USA) was distilled prior to use. Trifluoroacetic acid (TFA) (Alfa Aesar, Ward Hill, MA, USA) was used as received. Phosphate buffer (pH 7.4, Sigma Aldrich, St. Louis, MO, USA) was diluted with distilled water, 1:10, and 0.2 g/L sodium azide (NaN_3_) was added to prevent bacterial growth. Solvents for solubility test: Methanol (MeOH) (analytical grade, Avantor Performance Materials Poland S.A., Gliwice, Poland), acetonitrile (ACN) (for HPLC, Avantor Performance Materials Poland S.A., Gliwice, Poland), THF (analytical grade, Avantor Performance Materials Poland S.A., Gliwice, Poland), and chloroform (analytical grade, Avantor Performance Materials Poland S.A., Gliwice, Poland) were used as received.

### 2.2. Polymerization

The synthesis of block copolymers of glycidol and β-butyrolactone (PGl-b-P(β-BL)) and poly(ethylene glycol) methyl ether, glycidol, and β-butyrolactone (mPEG-b-PGl-b-P(β-BL)) was carried out according to the procedure described in [38]. In brief, as a representative example for PGl-b-P(β-BL), 10 mL of THF was transferred to a reactor containing 0.32 g (2.85 mmol) of *t*-BuOK. After dissolution of the initiator, 5.0 g (34.25 mmol) of freshly distilled EEGE was added. Polymerization was conducted at 55 °C. When the desired conversion of EEGE was reached, a solution of 0.308 g (3.08 mmol) of succinic anhydride in 10 mL of THF (1.08 equivalents relative to the alkoxide groups) was added. The reaction was stirred at room temperature for 2 h. Subsequently, 14.23 g (165.3 mmol) of freshly distilled β-BL was added, followed by 0.918 g (2.85 mmol) of TBA Br dissolved in 5 mL of DMSO. The reaction continued at room temperature until the desired β-BL conversion was achieved. Monomer conversion was monitored by ^1^H NMR based on the integration of signals corresponding to methyl protons of the polymer and monomer. Upon completion, solvents were removed under reduced pressure, and the copolymer was dried under high vacuum. For the synthesis of mPEG-b-PGl-b-P(β-BL), 10 mL of THF was added to the reactor containing 3.18 g (1.37 mmol) of mPEG-based macroinitiator. After dissolution of mPEG, 5.0 g (34.25 mmol) of freshly distilled EEGE was introduced into the reaction mixture. The mixture was heated in an oil bath at 55 °C until EEGE polymerization was complete (monitored by gas chromatography). Next, 0.148 g (1.48 mmol) of succinic anhydride in 10 mL of THF (1.08 equivalents relative to the mPEG hydroxyl groups) was added, and the reaction was carried out at room temperature for 2 h. Then, 5.3 g (61.65 mmol) of β-BL and 0.442 g (1.309 mmol) of TBA Br in 5 mL of DMSO were added, and copolymerization proceeded at room temperature until the desired conversion was achieved. Solvents were removed under reduced pressure, and the resulting copolymers were dried under high vacuum. Polymerization of β-BL, 5 g (58.08 mmol) of freshly distilled β-BL, and 10 mL of DMSO were transferred to the reactor containing 0.126 g (0.418 mmol) of tetrabutylammonium acetate. The reaction mixture was stirred at room temperature until full conversion of the monomer was achieved (^1^H NMR). Then, the obtained product was precipitated in deionized water and dried under reduced pressure.

### 2.3. Hydrolysis of Acetal Groups in Block Copolymers

To obtain copolymers with a PGl block, the acetal groups of the protected hydroxyl groups were removed by hydrolysis with TFA at room temperature under an argon atmosphere. Block copolymers were dissolved in a THF:water mixture (1:4 *v*/*v*), and TFA was added dropwise (10 mol% relative to the acetal groups of the PEEGE block). The progress of hydrolysis was monitored by ^1^H NMR by observing the disappearance of signals corresponding to EEGE units while simultaneously following the signals originating from the P(β-BL) block. After completion, solvents were removed under reduced pressure, and the copolymers were dried under high vacuum. Copolymers were designated PGl*_x_*-b-P(β-BL)*_y_* or mPEG_50_-b-PGl*_x_*-b-P(β-BL)*_y_*, where *x* and *y* denote the average degrees of polymerization of the respective blocks, and the value 50 refers to the average degree of polymerization of the mPEG-based macroinitiator.

### 2.4. Solubility Test

The solubility of the obtained block copolymers was tested by placing 20 mg of each copolymer into separate vials, followed by the addition of 2.5 mL of the respective solvent (*c* = 0.8%). The solubility test was performed at room temperature, and the dissolution progress was evaluated after 15 min. DP_phil_/D_phob_ was calculated as the ratio of hydrophilic block(s) (PGl or/and mPEG) to hydrophobic block (P(β-BL)).

### 2.5. Preparation of (Co)polymer Films

Glass Petri dishes (ø 60 mm, DUROPLAN, DWK Life Sciences, Wertheim, Germany) and silicon wafers (Cemat Silicon S.A., Bydgoszcz, Poland; with a 3 nm SiO_2_ layer) were first degreased with acetone, rinsed with distilled water and filtered ethanol, and then dried under reduced pressure. Copolymer solutions were prepared by dissolving 0.7 g of the respective copolymer in 15.5 mL of chloroform. The solutions were deposited using a syringe: 0.15 mL onto silicon wafers and 15 mL into Petri dishes. All samples were dried under reduced pressure until a constant weight was obtained.

### 2.6. Hydrolytic Degradation Study

Hydrolytic degradation of the copolymers was examined in phosphate buffer. (Co)polymer films deposited on Petri dishes were weighed and immersed in 9 mL of phosphate buffer (pH 7.4), followed by incubation at 37 °C. Degradation was evaluated after 1, 3, 5, 8, 12, 16 and 20 weeks. For solid samples, degradation was determined gravimetrically. At each time point, the buffer was removed, and the samples were rinsed with deionized water and dried to constant weight. The dry mass was recorded, and the samples were then transferred into fresh buffer. For samples forming emulsions, gravimetric analysis was not possible. Degradation was monitored by withdrawing a 100 µL aliquot of the sample at each time point, drying it under reduced pressure and analyzing it using SEC-MALLS.

### 2.7. Methods

The ^1^H NMR spectra were recorded in CDCl_3_ and DMSO-d_6_ using a Bruker Ultrashield 600 spectrometer (600 MHz, Billerica, MA, USA).

The molar masses of copolymers were determined using an SEC-MALLS system equipped with a multi-angle laser light scattering detector (DAWN HELEOS from Wyatt Technologies, Santa Barbara, CA, USA) and a refractive index detector (SEC-3010 WGE Dr. Bures, Dallgow-Doeberitz, Germany). The following column set system was used: guard GRAM 10µ; GRAM 100 Å; GRAM 1000 Å and GRAM 3000 Å (Polymer Standards Service PSS, Mainz, Germany). Measurements were carried out in DMF with 5 mmol/L LiBr at 45 °C with a nominal flow rate of 1 mL/min. The results were analyzed using ASTRA 7 software (Wyatt Technologies, Santa Barbara, CA, USA).

The water contact angle (*Θ*) was measured on dry silicon surfaces coated with the (co)polymers. Measurements were performed using a CAM101 goniometer (KSV Instruments, Helsinki, Finland) equipped with a camera (640 × 480 pixels) at room temperature. A series of images of a water droplet (~4 µL) was recorded over 30 s, and the average contact angle was calculated. Data analysis was conducted using the CAM2008 software.

Thermal analysis of the (co)polymers was carried out using a differential scanning calorimeter (DSC) TA Q2000 (TA Instruments, New Castle, DE, USA) under nitrogen (flow rate: 50 mL/min). Samples of approximately 10 mg were placed in aluminum pans and heated from −80 to +200 °C at a heating rate of 10 °C/min. The samples were then quenched with liquid nitrogen, followed by a second heating run to +200 °C. The melting point (*T*_m_) was determined from the first heating curve, and the enthalpy of melting and crystallization (Δ*H*) was calculated as the area under the peak, defined by the sigmoidal baseline. The glass transition temperature (*T*_g_) was determined as the inflection point of the second heating curve. The results were collected and analyzed using the Universal Analysis 2000 software.

Surface morphologies of the films were visualized using scanning electron microscopy (SEM) (Quanta 250 ESEM FEG, ThermoFischer Scientific, Waltham, MA, USA) with a large field detector (LFD) under a low vacuum mode (80 Pa) at an accelerating voltage of 10 kV.

## 3. Results and Discussion

### 3.1. Hydrolysis of Acetal Groups in the Block Copolymers

The block copolymers investigated in this study are composed of hydrophilic polyethers; mPEG and PGl blocks; and a hydrophobic, degradable polyester P(β-BL) segment. Diblock copolymer PGl-b-P(β-BL) and triblock copolymer mPEG-b-PGl-b-P(β-BL) of different compositions were synthesized *via* anionic ring-opening polymerization directly in a one-pot reaction. The synthesis followed a recently published method developed by the authors of [38], which employs a novel approach involving the quantitative transformation of alkoxide active centers, characteristic of oxirane polymerization, into carboxylate ones essential for the controlled anionic polymerization of β-BL.

**Scheme 1:**
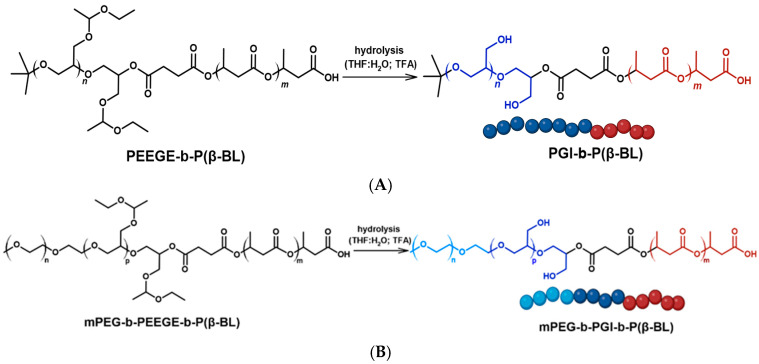
(**A**) Hydrolysis of acetal groups in diblock copolymers. (**B**) Hydrolysis of acetal groups in triblock copolymers.

To obtain a linear PGl block, its hydroxyl groups must be protected before polymerization. In this study, the hydroxyl groups of Gl were protected *via* the reaction of the monomer with ethyl vinyl ether, yielding 1-ethoxyethyl glycidyl ether containing an acetal group, which was subsequently employed in the synthesis of block copolymers. Typically, hydrolysis of acetal groups in PEEGE is carried out using two well-known approaches: a direct, single-stage method with hydrochloric acid or an indirect, two-stage method using formic acid [44], in which formate esters are first formed and then subjected to alkaline hydrolysis. However, the copolymers investigated here contain not only PGl and mPEG blocks but also P(β-BL) units with ester groups in the main chain, which are prone to hydrolysis. To address this issue, it was necessary to develop and optimize a mild hydrolysis procedure capable of selectively cleaving acetal groups without degrading the P(β-BL) segment. The hydrolysis of acetal groups in copolymer precursors was carried out in THF with varying water content, using different amounts of TFA. Complete deprotection of the acetal protecting groups was achieved using a mixture of THF:H_2_O (1:4 *v*/*v*) as a solvent and catalytic amounts of TFA at room temperature. The hydrolytic cleavage of acetal groups in the copolymers is depicted in Scheme 1A,B.

**Figure 1:**
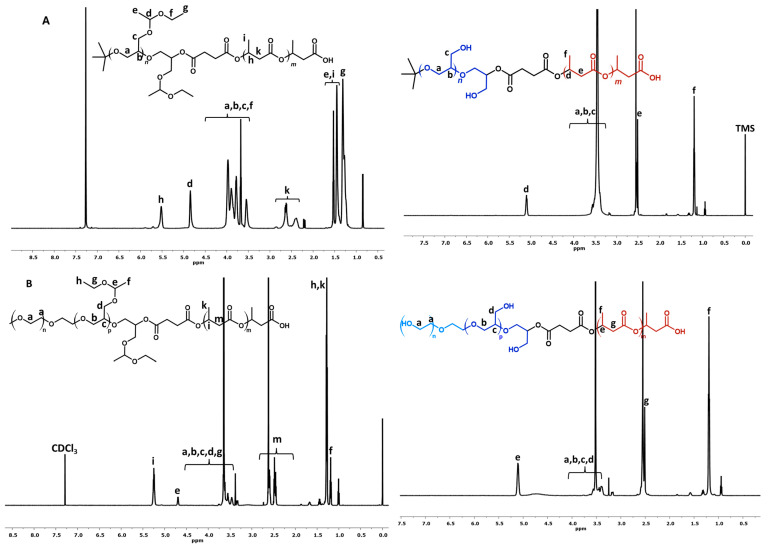
^1^H NMR of PEEGE_39_-b-P(β-BL)_30_ (**left**) and PGl_39_-b-P(β-BL)_30_ (**right**) (**A**); mPEG_50_-b-PEEGE_13_-b-P(β-BL)_63_ (**left**) and mPEG_50_-b-PGl_13_-b-P(β-BL)_63_ (**right**) (**B**) (600 MHz; CDCl_3_ and DMSO-d_6_).

The progress of hydrolysis was monitored using ^1^H NMR spectroscopy by observing the disappearance of signals corresponding to the EEGE units (methyl group protons at 1.20 ppm and the methine proton at 4.70 ppm) while simultaneously monitoring the signal of the methine proton of the P(β-BL) main chain at 5.10 ppm. These results confirm the complete hydrolysis of the acetal groups without degradation of the polyester block. Representative ^1^H NMR spectra of PEEGE_39_-b-P(β-BL)_30_; PGl_39_-b-P(β-BL)_30_; and mPEG_50_-b-PEEGE_13_-b-P(β-BL)_63_; mPEG_50_-b-PGl_13_-b-P(β-BL)_63_ are depicted in Figure 1; the other spectra of di- and triblock copolymers before and after hydrolysis are included in the Supplementary Materials (Figure S1).

Therefore, this approach enabled the controlled hydrolysis of acetal groups in the PEEGE block while preserving the integrity of ester groups in P(β-BL), demonstrating the feasibility of preparing polyether–polyester block copolymers. By combining these two approaches and straightforward synthesis followed by mild hydrolysis conditions, diblock PGl-b-P(β-BL) and triblock mPEG-b-PGl-b-P(β-BL) copolymers were obtained. These copolymers were designated as PGl*_x_*-b-P(β-BL)*_y_* and mPEG_50_-b-PGl_x_-b-P(β-BL)*_y_*, where *x* and *y* represent the average degrees of polymerization of the respective blocks, and the value 50 corresponds to the average degree of polymerization of the mPEG-based macroinitiator used for the synthesis (see Table 1).

Di- and triblock copolymers were subsequently used for the investigation of their properties, with particular emphasis on how the length and composition of individual blocks affect their overall properties and potential suitability for further applications.

### 3.2. Thermal Properties of PGl-b-P(β-BL) and mPEG-b-PGl-b-P(β-BL) Copolymers

**Figure 2:**
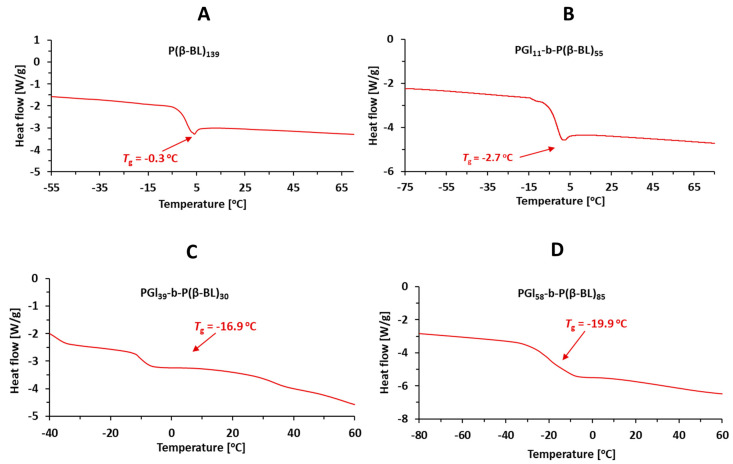
DSC curves of P(β-BL)_139_ (**A**) and PGl_11_-b-P(β-BL)_55_ (**B**); PGl_39_-b-P(β-BL)_30_ (**C**); PGl_58_-b-P(β-BL)_85_ (**D**).

Thermal properties of the obtained polymers were investigated using differential scanning calorimetry (DSC). Initially, the behavior of the synthesized P(β-BL)_139_ homopolymer was examined. The analysis revealed the amorphous character of the homopolymer, as evidenced by the absence of a melting transition in the thermogram, which is in agreement with literature reports [36]. Only a glass transition temperature *T*_g_ was detected at −0.3 °C (Figure 2A). Subsequently, the effect of incorporating hydrophilic PGl and mPEG segments into the P(β-BL) on the thermal properties of the resulting copolymers was evaluated. The *T*_g_ values for PGl-b-P(β-BL) and mPEG-b-PGl-b-P(β-BL) copolymers are summarized in Table 2.

Among the hydrophilic polyether segments, PGl is amorphous, with its glass transition temperature dependent on molar mass [45]. For degrees of polymerization comparable to those investigated in this study, the *T*_g_ of PGl is approximately −15 °C [45]. Accordingly, a decrease in *T*_g_ was anticipated for the PGl-b-P(β-BL) diblock copolymers relative to the P(β-BL) homopolymer. Indeed, incorporation of the PGl segment resulted in a reduction in the glass transition temperature with values ranging from −2.7 to −19.9 °C (Table 2), with the lowest *T*_g_ observed for the diblock copolymer containing the longest PGl block. The presence of a single glass transition temperature for all samples suggests good miscibility and compatibility between the blocks under the investigated conditions. DSC thermograms of the diblock copolymers are presented in Figure 2B–D.

In contrast, PEG is a semicrystalline polymer characterized by a significantly lower *T*_g_ than PGl [45]. The presence of this segment in the triblock copolymers resulted in a further decrease in *T*_g_, reaching values between −32.3 and −38.9 °C (Table 2).

DSC thermograms of the triblock copolymers revealed an exothermic peak (*T*_c_ = −2.6 °C, Δ*H* = 47.6 J/g) and an endothermic peak (*T*_m_ = 48 °C, Δ*H* = 47.7 J/g), attributed to crystallization and melting, respectively. Despite the predominance of amorphous PGl and P(β-BL) segments in the copolymer composition, the PEG crystalline contribution prevailed. An example DSC thermogram for the triblock mPEG_50_-b-PGl_24_-b-P(β-BL)_44_ is presented in the Supporting Information (Supplementary Materials, Figure S2).

Overall, the results indicate that the thermal behavior of the synthesized di- and triblock copolymers is strongly governed by both the composition and the architecture of the individual polymer segments.

### 3.3. Solubility in Different Solvents and Affinity to Water of PGl-b-P(β-BL) and mPEG-b-PGl-b-P(β-BL) Block Copolymers

The introduction of hydrophilic mPEG and PGl segments into the hydrophobic P(β-BL) backbone is expected to influence solubility, particularly in polar and semi-polar solvents and water affinity. In this context, the solubility of diblock PGl-b-P(β-BL) and triblock mPEG-b-PGl-b-P(β-BL) copolymers, varying in block lengths and composition, was systematically examined in different solvents, followed by an assessment of the water affinity of the resulting copolymer films. To better understand the effect of composition and block length, corresponding homopolymers with comparable degrees of polymerization were also tested, providing a reference that emphasizes the influence of structural organization on both solubility and water affinity. These results allow for a direct comparison of how the incorporation of hydrophilic polyether segments and variations in block architecture modify solubility behavior and water interactions, which is crucial for predicting the copolymers’ applicability in various processing conditions and potential applications.

The solubility of the samples was evaluated by dissolving 20 mg of each sample in 2.5 mL of the appropriate solvent (*c* = 0.8 wt%) at room temperature. The results are summarized in Table 3.

Due to the presence of hydroxyl groups and the resulting formation of hydrogen bonds, PGl requires highly polar solvents for dissolution, or solvents that also form hydrogen bonds or can act as hydrogen bond acceptors. In contrast, mPEG chains do not form hydrogen bonds between themselves and are therefore better solvated even by less polar solvents. The hydrophobic P(β-BL) is soluble in highly polar and highly solvating DMSO and in moderately polar solvents (ACN and THF) that can compete with the hydrophobic interactions between the segments of the P(β-BL) chains. For copolymers with a DP_phil_/DP_phob_ ratio greater than 1, insolubility is observed in water and moderately polar solvents, whereas dissolution occurs in strongly hydrogen-bonding and highly solvating solvents, such as DMSO and MeOH. Even when the hydrophobic P(β-BL) block predominates (DP_phil_/DP_phob_ = 0.68), the ability of DMSO and MeOH to participate in hydrogen-bonding interactions and effectively solvate the polymer chains outweighs the hydrophobic interactions between the P(β-BL) segments, thereby maintaining copolymer solubility. When the hydrophobic fraction is very high (DP_phil_/DP_phob_ = 0.20), the solubility of the copolymer is similar to that of P(β-BL); however, the presence of a small number of PGl units causes the copolymer to dissolve even in MeOH, despite the fact that P(β-BL) is insoluble in this solvent.

The solubility of triblock copolymers is different: the addition of mPEG units makes them soluble in all solvents used, including water. However, when the number of hydrophobic and hydrophilic units is similar, the copolymer becomes insoluble in water. This is likely due to the predominance of hydrophobic interactions between P(β-BL) units over the interactions between water molecules and mPEG and PGl units.

An analysis of the relationships between the structure of the copolymers studied and their solubility reveals that careful adjustment of the block composition is essential for controlling the interactions between the copolymers and the solvent, which significantly affects their solubility and potential applications.

The second parameter examined in this section was the water affinity of PGl-b-P(β-BL) and mPEG-b-PGl-b-P(β-BL), assessed by measuring the contact angle (*Θ*) of copolymer films deposited on silicon substrates. The films were prepared by casting 3% (*w*/*v*) solutions of the (co)polymers in chloroform onto silicon surfaces and drying under reduced pressure. The results are presented in Table 4.

**Figure 3:**
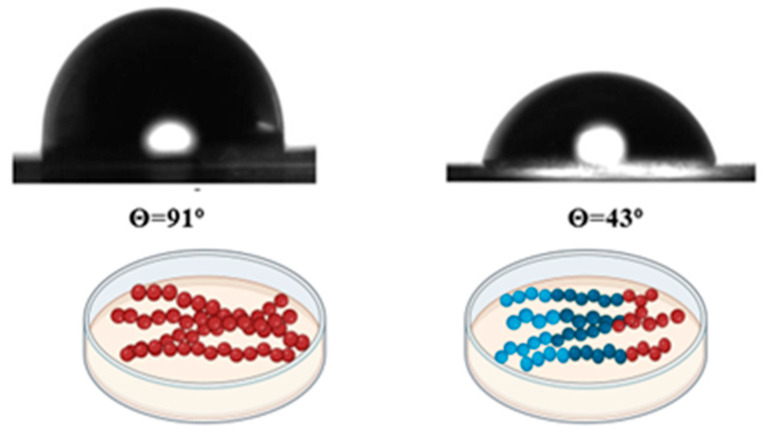
Water droplets on a silicone surface: P(β-BL)_139_ (**left**) and mPEG_50_-b-PGl_24_-b-P(β-BL)_44_ (**right**).

As summarized in Table 4, the contact angles of mPEG and PGl are ~20° [46] and <10° [47], respectively, confirming that both polymers are highly hydrophilic. The contact angles of P(β-BL) films were relatively high, *Θ* = 91°, reflecting their hydrophobic nature, whereas the incorporation of hydrophilic PGl segments slightly decreased the value of the contact angle, indicating increased surface hydrophilicity. The contact angles of the obtained diblock copolymers, PGl_11_-b-P(β-BL)_55_, PGl_39_-b-P(β-BL)_30_ and PGl_58_-b-P(β-BL)_85_, are governed by the balance between the hydrophilic PGl block and the hydrophobic P(β-BL) block. The most pronounced reduction in *Θ* was observed for triblock copolymers containing mPEG. This trend is in good agreement with the solubility tests described above. Representative images of water droplets deposited on surfaces coated with the films of the investigated samples are shown in Figure 3.

In any case, the introduction of a hydrophilic moiety (mPEG or PGl) into P(β-BL) significantly reduces the contact angle, thereby improving the wettability of the surfaces prepared from the copolymers investigated. It is evident that incorporating hydrophilic polyether blocks into the hydrophobic P(β-BL) backbone modifies the surface properties of this polyester in a manner dependent on the relative contribution of each block. These results indicate that the surface hydrophilicity of the copolymers can be tuned through block composition, which is important for applications requiring controlled wettability.

### 3.4. Hydrolytic Degradation Studies of the P(β-BL) Block in PGl-b-P(β-BL) and mPEG-b-PGl-b-P(β-BL) Copolymers

**Scheme 2:**
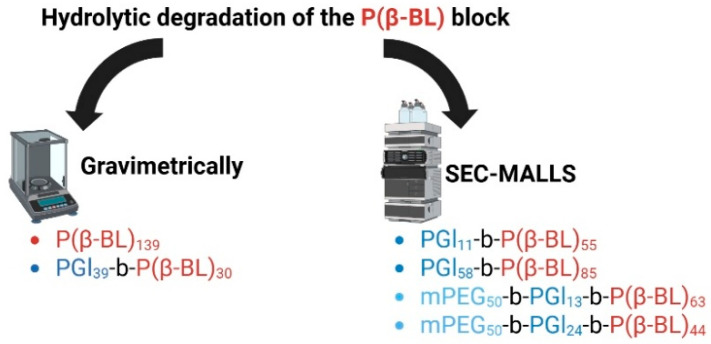
Methods for studying the hydrolytic degradation of the P(β-BL) in di- and triblock copolymers.

An important factor in the design of materials based on degradable polymers for potential biomedical applications is the ability to control their degradation. P(β-BL) is a hydrophobic polymer, and its degradation is typically slow and poorly controlled. Therefore, a key objective of this study was to investigate the effect of incorporating hydrophilic mPEG and PGl blocks on the rate and controllability of this process. Consequently, the synthesized diblock PGl-b-P(β-BL) and triblock mPEG-b-PGl-b-P(β-BL) copolymers were subjected to hydrolytic degradation studies of the P(β-BL) segment. The degradation of both di- and triblock copolymers was studied in the form of films cast onto Petri dishes and immersed in phosphate buffer. Due to differences in the composition of the investigated copolymers and in the content of hydrophilic blocks, resulting in distinct solubility behaviors, the hydrolytic degradation of the studied di- and triblock copolymers was evaluated using two methods, as illustrated in Scheme 2.

**Figure 4:**
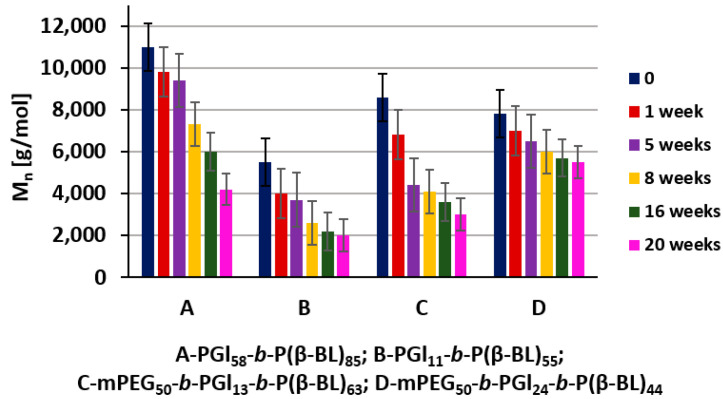
Changes in molar mass of di- and triblock copolymers during the degradation process.

Degradation of the diblock copolymers PGl_11_-b-P(β-BL)_85_ and PGl_58_-b-P(β-BL)_85_, as well as the triblock copolymers mPEG_50_-b-PGl_13_-b-P(β-BL)_63_ and mPEG_50_-b-PGl_24_-b-P(β-BL)_44_, which formed emulsions in phosphate buffer, was analyzed using SEC-MALLS by monitoring the decrease in molar mass with time. In contrast, for P(β-BL)_139_ and PGl_39_-b-P(β-BL)_30_, which remained insoluble in phosphate buffer, degradation was monitored gravimetrically by measuring sample weight loss over time. This dual approach enabled a comparative evaluation of degradation behavior across all investigated samples. It should be noted that the applied analytical methods provide complementary information regarding the degradation process and therefore do not allow for direct quantitative comparison of degradation rates between all samples. SEC-MALLS monitors changes in molar mass associated with hydrolytic degradation of the P(β-BL) block, which leads to a chain scission occurring at the early stages of hydrolysis, whereas gravimetric analysis reflects macroscopic mass loss. The selection of the analytical method was dictated by the different solubility of the investigated samples, which prevented reliable evaluation of all copolymers using a single technique. Therefore, the obtained results were interpreted primarily in terms of overall degradation tendencies and qualitative differences in degradation behavior. Hydrolytic degradation was performed in phosphate buffer (pH 7.4) at 37 °C to mimic physiological conditions, and the samples were incubated for up to 20 weeks. The molar mass changes of the di- and triblock copolymers during hydrolytic degradation are presented in Figure 4.

As shown in the presented diagram, over 20 weeks of hydrolytic degradation, the molar mass of the examined di- and triblock copolymers decreased compared to their initial values. For the diblock copolymers PGl_11_-b-P(β-BL)_55_ and PGl_58_-b-P(β-BL)_85_, as well as the triblock copolymer mPEG_50_-b-PGl_13_-b-P(β-BL)_63_, the molar mass decreased by approximately 60% during the degradation process. In contrast, the triblock copolymer mPEG_50_-b-PGl_24_-b-P(β-BL)_44_ exhibited a 30% reduction in molar mass. Both mPEG and PGl are resistant to hydrolytic degradation; therefore, the observed changes can be attributed solely to the hydrolytic cleavage of ester bonds in the P(β-BL) segment. The most pronounced molar mass loss was observed in copolymers containing the highest content of the P(β-BL) unit. In particular, the molar mass of mPEG_50_-b-PGl_13_-b-P(β-BL)_63_ decreased from *M*_n_ = 8 600 g/mol to *M*_n_ = 3000 g/mol; therefore, it can be concluded that the polyester block was degraded, leaving predominantly polyether segments in this copolymer. In mPEG_50_-b-PGl_13_-b-P(β-BL)_63_, the presence of both mPEG and PGl segments appears to accelerate the hydrolytic degradation of the polyester block, likely due to enhanced accessibility of water to the ester bonds.

**Figure 5:**
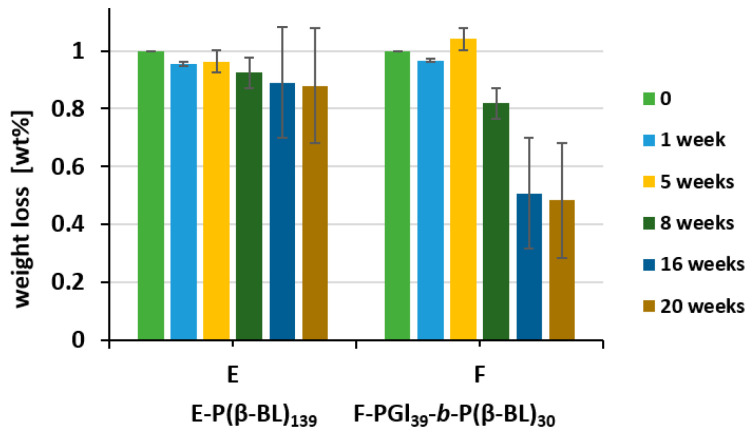
Changes in weight sample of P(β-BL)_139_ (E) and PGl_39_-b-P(β-BL)_30_ (F) during hydrolytic degradation process.

The hydrolytic degradation of PGl_39_-b-P(β-BL)_30_ and P(β-BL)_139_ was evaluated gravimetrically by measuring the sample weight loss over time. During the early stages of degradation, an increase in sample weight was observed, particularly for the diblock copolymer, while the P(β-BL)_139_ showed a smaller weight gain (Figure 5).

This behavior can be attributed to the presence of the hydrophilic PGl block attached to the hydrophobic P(β-BL) chain, which enhanced the wettability of the copolymer and, consequently, increased water absorption during the initial stage of the experiment. In the subsequent weeks, significant weight loss was observed only for PGl_39_-b-P(β-BL)_30_, whereas the P(β-BL)_139_ sample exhibited a much smaller weight reduction.

The progress of hydrolytic degradation of the P(β-BL) segment in PGl-b-P(β-BL) and mPEG-b-PGl-b-P(β-BL) block copolymers was also evaluated by measuring the water contact angles of copolymer films coated on silicone substrates after 20 weeks of exposure to phosphate buffer at 37 °C. For clarity, the water contact angle values measured prior to hydrolytic degradation are also included to facilitate comparison. These data were obtained from earlier measurements of the water affinity of the synthesized di- and triblock copolymers. The results of water contact angle measurements before and after hydrolytic degradation are shown in Table 5.

**Figure 6:**
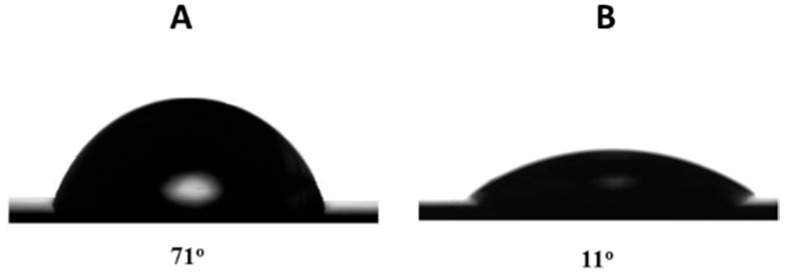
Water droplets on silicon surface coated with PGl_39_-b-P(β-BL)_30_ before (**A**) and after (**B**) hydrolytic degradation.

Water contact angle measurements for the di- and triblock copolymers after hydrolytic degradation ranged from 11° to 5°. The marked increase in surface wettability compared to the initial values is attributed to the degradation of the hydrophobic P(β-BL) block, which reduces its relative contribution to the overall copolymer composition. Figure 6 illustrates a water droplet on a silicon surface coated with PGl_39_-b-P(β-BL)_30_ before and after the degradation process, highlighting the changes in surface hydrophilicity. For the β-BL homopolymer, a decrease in contact angle was also observed, although it was less pronounced than in the case of the polyether–polyester block copolymers.

Therefore, the results indicate that incorporating hydrophilic mPEG and PGl blocks into the P(β-BL) chain enhanced surface wettability, thereby affecting the degradation rate of the polyester [23,37,48,49].

**Figure 7:**
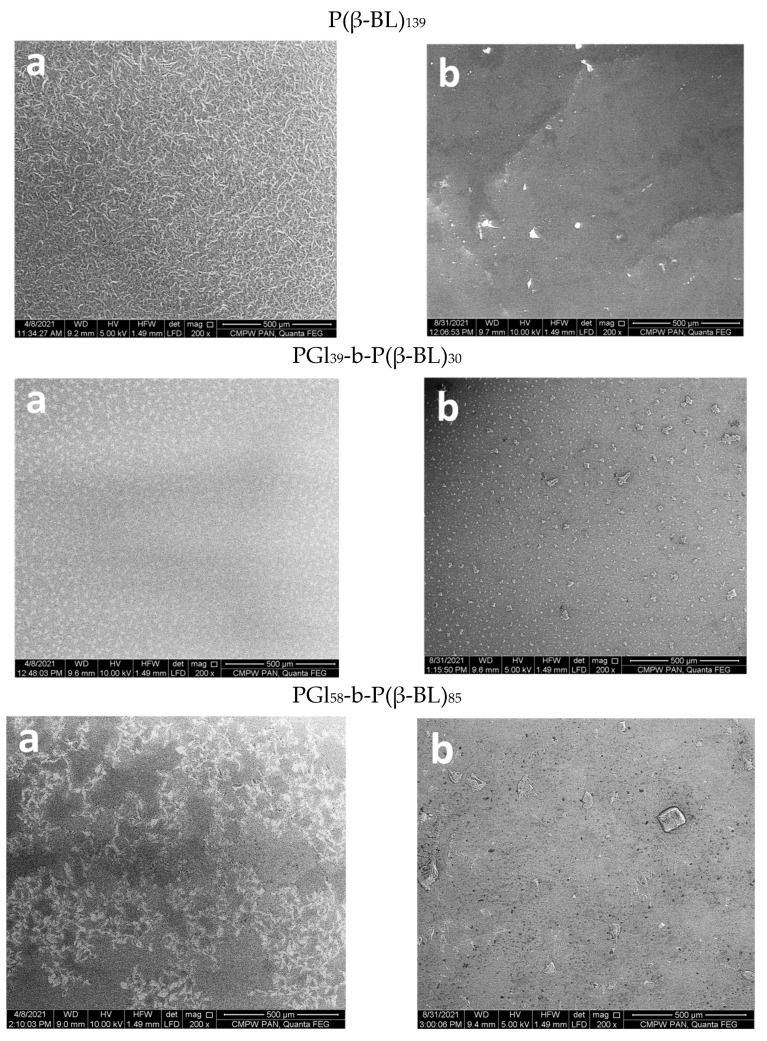

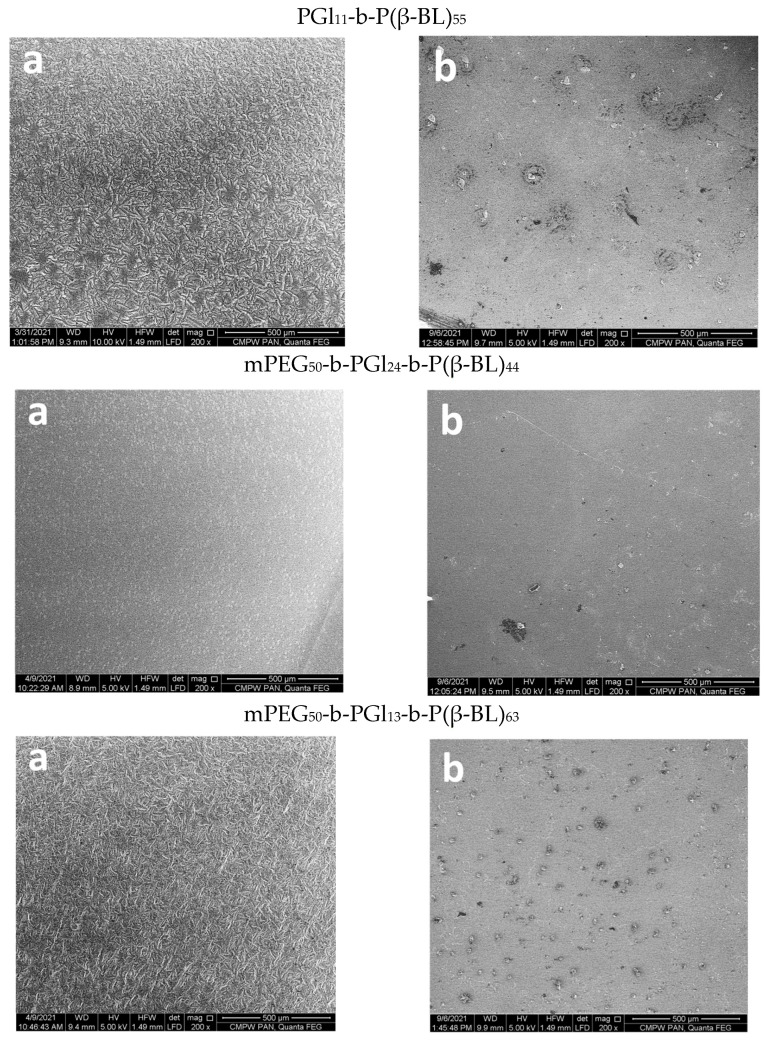
Surface morphology (SEM) of P(β-BL) and di- and triblock-copolymer-coated films before (**a**) and after (**b**) hydrolytic degradation.

In addition, the surface morphology of the samples before and after degradation was examined by SEM to assess changes in the copolymer films induced by hydrolytic degradation. The morphology of P(β-BL)-, PGl-b-P(β-BL)-, and mPEG-b-PGl-b-P(β-BL)-coated films is presented in Figure 7, providing further support for the degradation study results.

SEM images reveal that prior to degradation, all samples exhibit textured surfaces without phase separation. After hydrolytic degradation, the surfaces become more flattened and uniform, whereas di- and triblock copolymer surfaces remain relatively smooth, with small holes visible compared to the P(β-BL) homopolymer. These differences are likely associated with the higher wettability of surfaces containing hydrophilic mPEG and PGl segments, which promotes water penetration and accelerates hydrolysis of the P(β-BL) block. In contrast, the hydrophobic P(β-BL) homopolymer restricts water access, resulting in slower chain cleavage. Overall, the morphological observations support the degradation study results, indicating that incorporation of hydrophilic polyether mPEG and PGl blocks enhances the hydrolytic degradation of P(β-BL) by improving surface wettability and facilitating water penetration into the polymer matrix.

## 4. Conclusions

Di- and triblock copolymers of PGl-b-P(β-BL) and mPEG-b-PGl-b-P(β-BL) were synthesized *via* a one-pot approach, and a selective mild hydrolysis method was developed to remove acetal-protecting groups from the PGl block without affecting the P(β-BL) segment. The thermal properties of the copolymers were found to depend on block composition and length, with *T*_g_ values ranging from −2.7 to −19.9 °C for diblock copolymers and with mPEG-containing triblock copolymers exhibiting particularly lower glass transition temperatures up to −38.9 °C. The presence of a single glass transition temperature for all copolymers suggests the good compatibility of the constituent blocks and indicates limited phase separation under the investigated conditions. The incorporation of hydrophilic PGl and mPEG blocks enhanced solubility and surface wettability, which, in turn, accelerated the hydrolytic degradation of the hydrophobic P(β-BL) segment. Compared to the hydrophobic P(β-BL) film, the water contact angle decreased to 53° for the PGl-b-P(β-BL) copolymers and further decreased to 43° for the mPEG-b-PGl-b-P(β-BL) copolymers, indicating enhanced surface hydrophilicity. The most significant decrease in molar mass during degradation, exceeding 50% relative to the initial non-degraded sample, was observed for the diblock copolymers, whereas the P(β-BL) sample exhibited only a minor reduction of a few percent. Surface characterization, including morphological analysis and water contact angle measurements, confirmed changes consistent with increased water accessibility. These findings demonstrate that precise tuning of block composition and architecture enables control over thermal properties, solubility, and degradation behavior, highlighting the potential of these copolymers for biomedical applications, such as drug delivery systems and degradable coatings.

## Figures and Tables

**Table 1:** Di- and triblock copolymers obtained after hydrolysis of acetal groups.

Diblock Precursors	Diblock Copolymers	Triblock Precursors	Triblock Copolymers
PEEGE_11_-b-P(β-BL)_55_	PGl_11_-b-P(β-BL)_55_	mPEG_50_-b-PEEGE_13_-b-P(β-BL)_63_	mPEG_50_-b-PGl_13_-b-P(β-BL)_63_
PEEGE_39_-b-P(β-BL)_30_	PGl_39_-b-P(β-BL)_30_	mPEG_50_-b-PEEGE_24_-b-P(β-BL)_44_	mPEG_50_-b-PGl_24_-b-P(β-BL)_44_
PEEGE_58_-b-P(β-BL)_85_	PGl_58_-b-P(β-BL)_85_		

**Table 2:** Glass transition temperature of the homo- and copolymers.

Homopolymers	*T*_g_ [°C]	Diblock Copolymers	*T*_g_ [°C]	Triblock Copolymers	*T*_g_ [°C]
mPEG_50_	<−60 [45]	PGl_11_-b-P(β-BL)_55_	−2.7	mPEG_50_-b-PGl_13_-b-P(β-BL)_63_	−32.3
PGl_55_	−15 [45]	PGl_39_-b-P(β-BL)_30_	−16.9	mPEG_50_-b-PGl_24_-b-P(β-BL)_44_	−38.9
P(β-BL)_139_	−0.3	PGl_58_-b-P(β-BL)_85_	−19.9		

**Table 3:** Solubility of homo- and copolymers in water and organic solvents.

Sample	DP_phil_/DP_phob_	Water	DMSO	ACN	MeOH	THF
PGl_48_		**+**	**+**	**−**	**+**	**−**
mPEG_50_		**+**	**+**	**+**	**+**	**+**
P(β-BL)_139_		**−**	**+**	**+**	**−**	**+**
PGl_39_-b-P(β-BL)_30_	1.30	**−**	**+**	**−**	**+**	**−**
PGl_58_-b-P(β-BL)_85_	0.68	**−**	**+**	**−**	**+**	**−**
PGl_11_-b-P(β-BL)_55_	0.20	**−**	**+**	**+**	**+**	**+**
mPEG_50_-b-PGl_24_-b-P(β-BL)_44_	1.68	**+**	**+**	**+**	**+**	**+**
mPEG_50_-b-PGl_13_-b-P(β-BL)_63_	1.00	**−**	**+**	**+**	**+**	**+**

“+” denotes solubility; “−” denotes insolubility.

**Table 4:** Water contact angles (*Θ*) for the homo- and copolymer films.

Homopolymers	Contact Angle *Θ* [°]	Diblock Copolymers	Contact Angle *Θ* [°]	Triblock Copolymers	Contact Angle *Θ* [°]
mPEG	~20 [46]	PGl_11_-b-P(β-BL)_55_	74 ± 1	mPEG_50_-b-PGl_13_-b-P(β-BL)_63_	66 ± 1
PGl	<10 [47]	PGl_39_-b-P(β-BL)_30_	71 ± 1	mPEG_50_-b-PGl_24_-b-P(β-BL)_44_	43 ± 1
P(β-BL)_139_	91 ± 1	PGl_58_-b-P(β-BL)_85_	53 ± 1		

**Table 5:** Water contact angles (*Θ*) for the homo- and copolymer films before (*t* = 0) and after (*t* = 20) hydrolytic degradation.

Homopolymer	Contact Angle *Θ* [°]		Diblock Copolymers	Contact Angle *Θ* [°]		Triblock Copolymers	Contact Angle *Θ* [°]	
	*t* = 0	*t* = 20		*t* = 0	*t* = 20		*t* = 0	*t* = 20
P(β-BL)_139_	91 ± 1	65 ± 1	PGl_11_-b-P(β-BL)_55_	74 ± 1	11 ± 1	mPEG_50_-b-PGl_13_-b-P(β-BL)_63_	66 ± 1	9 ± 1
			PGl_39_-b-P(β-BL)_30_	71 ± 1	11 ± 1	mPEG_50_-b-PGl_24_-b-P(β-BL)_44_	43 ± 1	<5 ± 1
			PGl_58_-b-P(β-BL)_85_	53 ± 1	<5 ± 1			

## Data Availability

The original contributions presented in this study are included in the article and Supplementary Materials. Further inquiries can be directed to the corresponding author.

## Supplementary Materials

The following supporting information can be downloaded at https://www.mdpi.com/article/10.3390/ma19122467/s1: Figure S1: ^1^H NMR of di- and triblock copolymers before and after hydrolysis (600 MHz; CDCl_3_ and DMSO-d_6_). Figure S2: DSC curves of triblock copolymers.
