# Supplementary material for: Block Copolymers Based on Ethylene Glycol, Glycidol and β-Butyrolactone with Tunable Thermal Properties, Solubility, and Hydrolytic Degradation
Source: Materials (Basel). 2026 Jun 9;19(12):2467. doi: 10.3390/ma19122467 (PMC13303053; doi:10.3390/ma19122467)
Supplement: Supplementary file 1 [file materials-19-02467-s001.zip › materials-4309273-supplementary.pdf]

Supplementary Information

Block Copolymers Based on Ethylene Glycol, Glycidol and β-Butyrolactone with Tunable Thermal Properties, Solubility, and Hydrolytic Degradation

Marcelina Bochenek *, Natalia Oleszko-Torbus, Agnieszka Kowalczuk and Wojciech Wałach

Centre of Polymer and Carbon Materials, Polish Academy of Sciences,
M. Curie-Skłodowskiej 34, 41-819 Zabrze, Poland
* Correspondence: mbochenek@cmpw-pan.pl

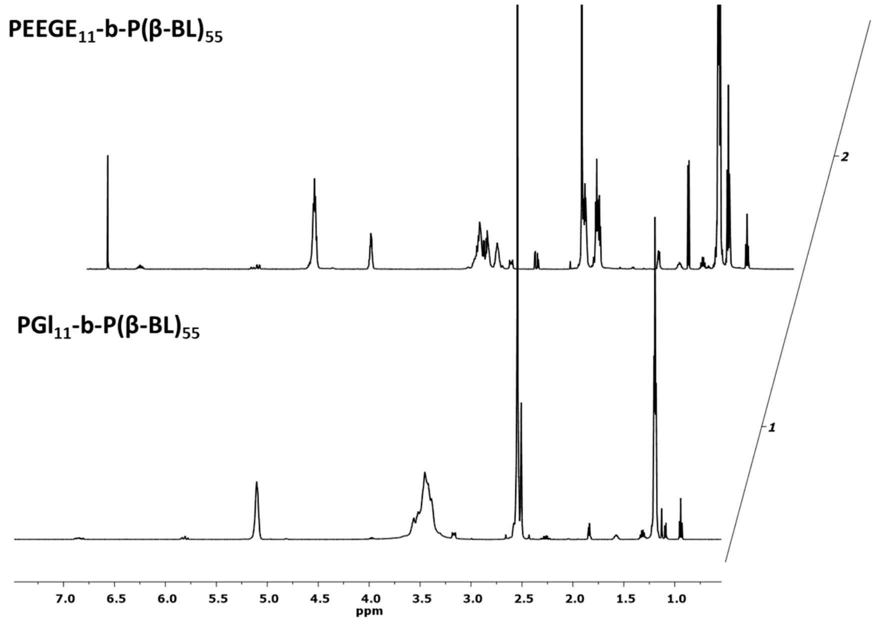

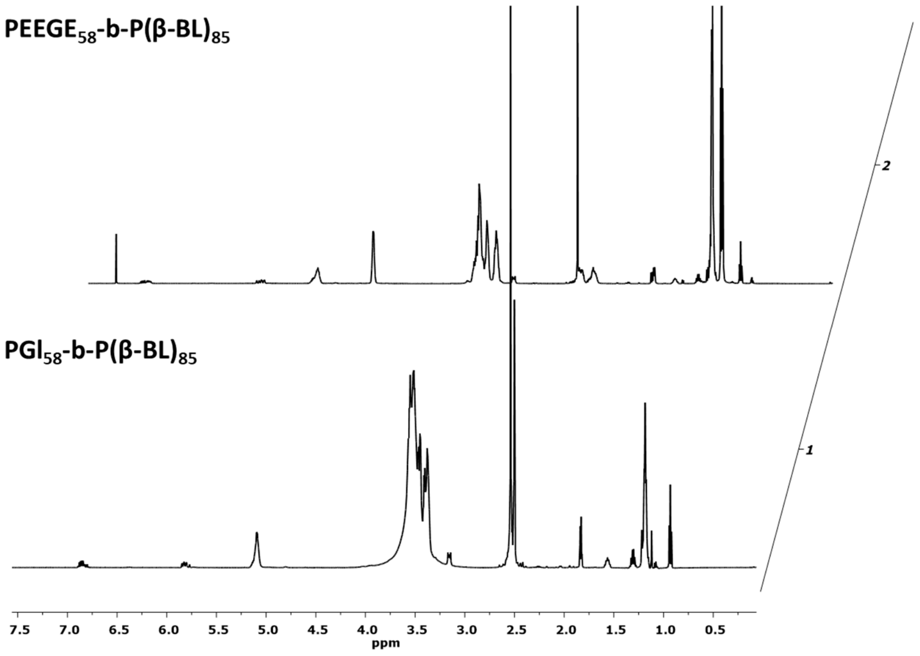

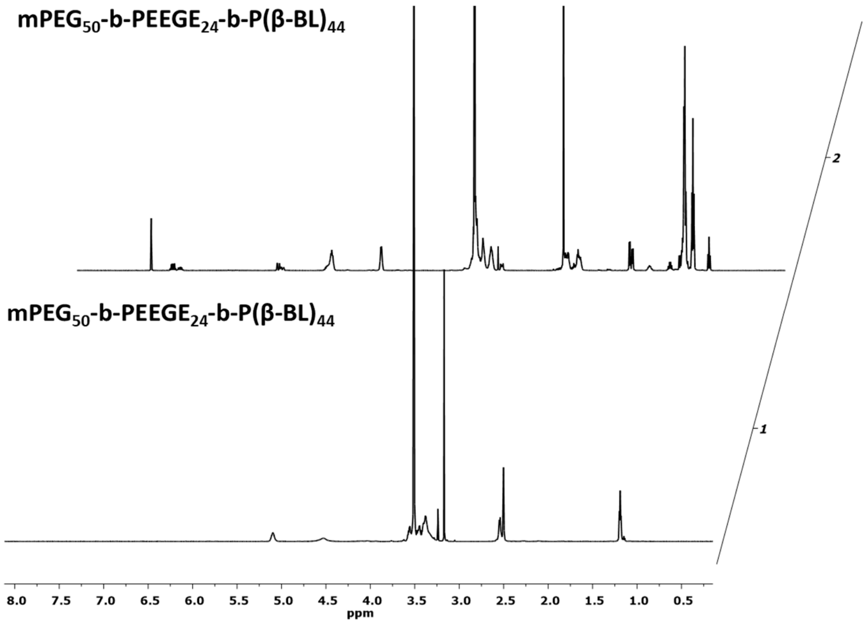

Figure S1. ^{1}H NMR of di- and triblock copolymers before and after hydrolysis (600 MHz; $CDCl_3$ and DMSO-d_6).

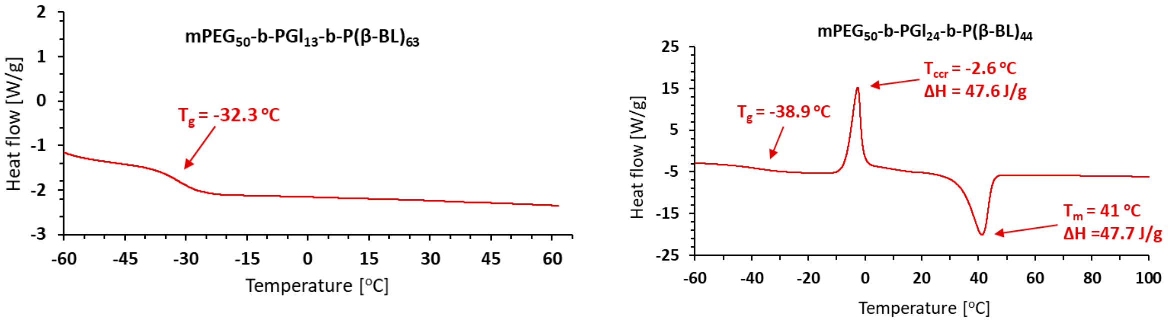

Figure S2. DSC curves of triblock copolymers.
